# The use of veno-veno-arterial ECMO as a successful strategy in acute mitral regurgitation secondary to papillary muscle rupture causing cardiogenic shock and profound hypoxemia: a case report

**DOI:** 10.1093/jscr/rjae408

**Published:** 2024-06-06

**Authors:** Nael Al-Sarraf, Adel Maher, Nanda Kishore Muddaiah, Yuldash Agzamov, Nawar Jabbour

**Affiliations:** Department of Cardiac Surgery, Dabbous Cardiac Center, Adan Hospital, Kuwait City, Kuwait; Department of Cardiac Surgery, Dabbous Cardiac Center, Adan Hospital, Kuwait City, Kuwait; Department of Anesthesia and Critical Care, Dabbous Cardiac Center, Adan Hospital, Kuwait City, Kuwait; Department of Anesthesia and Critical Care, Dabbous Cardiac Center, Adan Hospital, Kuwait City, Kuwait; Department of Anesthesia and Critical Care, Dabbous Cardiac Center, Adan Hospital, Kuwait City, Kuwait

**Keywords:** mitral regurgitation, extracorporeal membrane oxygenator, papillary muscle rupture, cardiogenic shock

## Abstract

Acute mitral regurgitation (MR) secondary to papillary muscle rupture is a rare mechanical complication of acute myocardial infarction occurring in 0.05–0.26% of all cases of myocardial infarction. The only treatment is emergency mitral valve surgery with high operative mortality reaching up to 39%. The use of extracorporeal membrane oxygenator (ECMO) as a stabilization strategy and a bridge to recovery may potentially improve the outcome of such cases. Here, we report a case of acute MR presenting with cardiogenic shock and severe hypoxia that required insertion of veno-veno-arterial ECMO initially and followed by emergency mitral valve replacement. This strategy proved useful with full recovery of the patient.

## Introduction

Acute mitral regurgitation (MR) secondary to papillary muscle rupture (PMR) carries a high mortality rate following emergency surgery. The use of extracorporeal membrane oxygenator (ECMO) can be of benefit in stabilizing these patients and bridge them for recovery. Here we report a case of acute MR that presented with cardiogenic shock and severe hypoxemia that required veno-veno-arterial ECMO (VVA ECMO) with emergency mitral valve replacement (MVR) that recovered fully postoperatively.

## Case report

**Figure 1:**
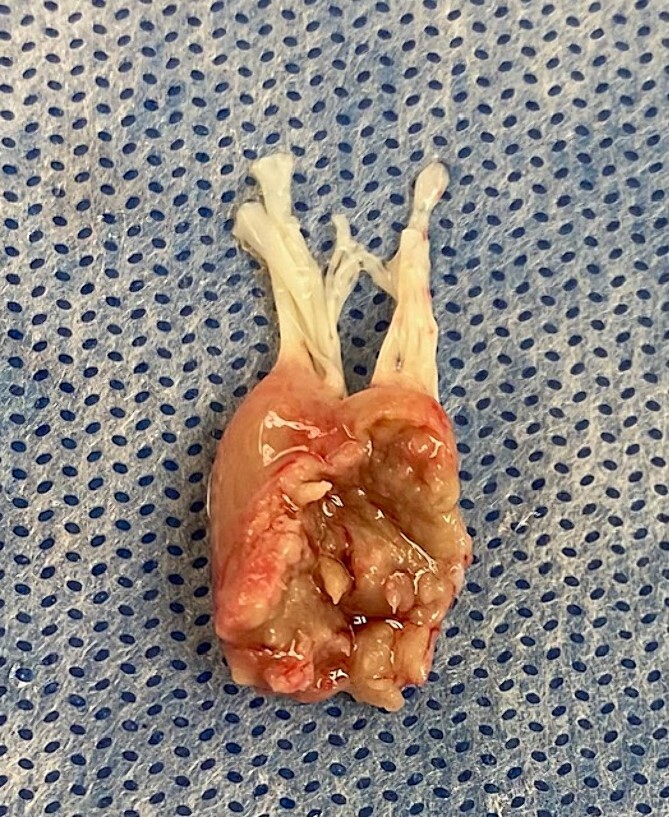
Intra-operative view of papillary muscle complete rupture.

A 39 years old previously healthy man presented with sudden onset chest pain and dyspnea. He was diagnosed as lateral wall ST elevation myocardial infarction (MI) with pulmonary edema. He sustained arrest and required intubation and ventilation. Transthoracic echocardiography showed severe acute MR secondary to anterolateral PMR. He underwent coronary angiography which showed acute occlusion of circumflex artery. An intra-aortic balloon pump (IABP) was inserted into the right femoral artery. At that time, there was difficulty in oxygenation of the patients with 100% oxygen requirement and high positive end expiratory pressure (PEEP). To help with this severe hypoxemia, veno-veno ECMO was inserted using left femoral vein and right internal jugular vein. Patient started to have recurrent ventricular arrhythmias with hypotension requiring inotropes and vasopressors. Then arterial cannula was inserted into left femoral artery with antegrade leg perfusion cannula and connected to the ECMO circuit as VVA ECMO. Flow was adjusted as 4 L VV ECMO and 1 L only as VA ECMO by using a partial clamp and most of this arterial flow was perfusing the leg. Levosimendan was started at 0.2 mcg/kg/min to improve forward flow and avoid left ventricular distension. Patient was taken immediately to operative room. Intra-operative trans-esophageal echocardiography (TEE) confirmed the severe MR. Patient underwent sternotomy with ascending aorta and bi-caval cannulation. Patient then placed on cardiopulmonary bypass (CPB) with ascending aorta cross clamped and a combination of antegrade and retrograde cold blood cardioplegia given. Saphenous vein graft was anastomosed to first obtuse marginal artery and mitral valve was then approached through superior trans-septal incision. Anterolateral PMR was found (Fig. 1) and mitral valve was replaced with size 29 St Jude mechanical valve with preservation of posterior leaflet and part of anterior leaflet at A3 level. Patient was weaned from CPB to the VVA ECMO at 4.2 L flow (2.7 L flow VV ECMO and 1.7 L VA ECMO). At the end of the operation, the patient was on dobutamine 10 mcg/kg/min, norepinephrine 0.02 mcg/kg/min, and epinephrine 0.05 mcg/kg/min. TEE showed well-functioning mitral valve prosthesis with no leak. As patient was coagulopathic, his sternum was left open and packed for 24 hours to prevent any tamponade and to control the bleeding sites. By Day 1 postoperatively, the ECMO flow was 4.2 L/min with minimum inotropic support (levosimendan 0.1 mcg/kg/min and dobutamine 5 mcg/kg/min). Patient was taken to operative room for sternal washout and closure. By that time, contractility of the heart was improving and arterial cannula with antegrade leg perfusion was removed, and sternum closed. The IABP was removed 2 hours later. As the chest x-ray was congested with pulmonary edema, patient was kept on VV ECMO only. By Day 2, his VV ECMO flow was reduced gradually to 2.5 L flow and his forced inspiratory oxygen (FiO2) was 50% with PEEP of 8. Patient maintained good urine output of 1.8 L/24 hours with reduction of his creatinine level (resolving acute kidney injury). He was on levosimendan 0.1 mcg/kg/min and dobutamine 5 mcg/kg/min. Lactate level was normal. By Day 4 postoperatively, his VV ECMO was removed and tracheostomy was performed. By Day 5, patient developed seizures activity and computerized tomography (CT) of brain showed no acute insult apart from chronic brain infarction and small vessel disease. Repeat CT brain 2 days later showed no changes, and the seizures were controlled with anticonvulsant therapy. He was kept in intensive care to wean the tracheostomy, and this was achieved by Day 21 postoperatively when he was sent to the ward. In the ward he continued to improve with physiotherapy and was fully mobile. He was discharged home directly by Day 35 postoperatively with therapeutic level of warfarin and clear chest x-ray with oxygen saturation of 100% on room air with totally independent level of activity. Pre-discharge echocardiography showed well-functioning mitral valve prosthesis with no para-valvular leak and mean gradient of 3 mmHg with left ventricular ejection fraction of 50%. Patient was seen in the outpatient clinic at 3 months following surgery and he was in sinus rhythm with normal kidney function and was back to his work.

## Discussion

Acute MR secondary to PMR is a rare mechanical complication of acute MI with an incidence of 0.05–0.26% [1]. The definite treatment remains emergency mitral surgery with a high operative mortality (24–39%) [2, 3]. During the acute phase of MR, there is an abrupt increase in left atrial (LA) pressure without LA adaptation which results in a significant increase in pulmonary venous pressure secondary to the acute volume overload. This results in severe pulmonary edema and hypoxia. At the same time, the cardiac output begins to drop due to reduced forward stroke volume across the aortic valve contributing to the cardiogenic shock physiology. This triggers a compensatory systemic vasoconstrictor response causing an increase in the afterload which further aggravates MR [4]. The rationale for using ECMO preoperatively is to maintain tissue perfusion and further prevents multiorgan failure until definitive surgical treatment is performed. These cases postoperatively require oxygenation support for the resultant pulmonary edema and hypoxia that will require lengthy time for the lungs to recover.

The largest study on the use of VA ECMO in PMR was a retrospective analysis of multicenter registry (the CAUTION study) [1]. The authors reported on 23 patients that required VA ECMO support (with 43.5% of patients requiring it preoperatively). The mean duration of ECMO support was 4 days and the complication rates occurred in half of the cases with in-hospital mortality of 39%. However, the authors used the conventional VA ECMO configuration as opposed to our case. The use of VVA ECMO has been reported in cases of respiratory failure that was initially started on conventional VA or VV ECMO and later re-configured to VVA ECMO due to various reasons [5]. They reported on 10 cases with survival rate at discharge of 50% including the ones that required a lung transplantation. The authors have changed the configuration of ECMO to VVA at a mean of 2 days following insertion of VV or VA ECMO.

In our case, the patient was in cardiogenic shock and severe hypoxemia secondary to severe pulmonary edema. The use of VVA ECMO configuration was particularly useful (both preoperatively and postoperatively) in conjunction with emergency MVR. This was shown by the early improvement in hemodynamic status within 24 hours of surgery which resulted in removal of arterial cannula of ECMO and keeping the ECMO as VV ECMO only for oxygenation with decannulation on Day 4 postoperatively. Few previous reports have reported the different timing of performing surgery following ECMO insertion [5, 6]. In addition, different approaches with the use of ECMO support were used such as VA ECMO [6, 7] or Tandem Heart and VVA ECMO combination [4].

## Conclusion

The use of VVA ECMO configuration together with emergency MVR in patients with acute MR secondary to PMR presenting as cardiogenic shock and hypoxemia can be used a successful strategy rather than using traditional ECMO configuration.
